# Dynamic Balance Control and Postural Adaptation in Human-Robot Collaborative Manipulation: Within-Subject Experimental Study

**DOI:** 10.2196/79930

**Published:** 2026-04-23

**Authors:** Alessia de Nobile, Daniele Bibbo, Simone Ranaldi, Maurizio Schmid, Giovanni Corvini, Silvia Conforto

**Affiliations:** 1 Department of Industrial, Electronic and Mechanical Engineering, Roma Tre University, Via Vito Volterra, 62 - Corpo B, Roma, 00146, Italy, 39 0657337298; 2 Biorobotics Group, Spanish National Research Council (CSIC) Center for Automation and Robotics (CAR) CSIC-UPM, Madrid, Spain

**Keywords:** human-robot collaboration, center of pressure, recurrence quantification analysis, biomechanical risk, ergonomics, HRC, COP, RQA

## Abstract

**Background:**

The integration of robots into industrial settings has rapidly advanced, aiming to reduce human involvement in demanding tasks while improving overall efficiency. As collaborative robots (cobots) become more prevalent, assessing the physical strain during joint tasks is essential to promote long-term well-being in the workplace.

**Objective:**

This study aimed to investigate how human-robot collaboration influences workers’ postural control and musculoskeletal load during manipulation tasks performed in parallel.

**Methods:**

Fourteen healthy male participants performed manipulation tasks under 3 conditions: without robotic assistance, with a cobot providing load support (Robot Free [RF]) and a cobot constrained to horizontal movement (Robot Plane [RP]). Center of pressure trajectories were computed, and nonlinear recurrence quantification analysis indicators (recurrence rate [REC], determinism [DET], and their ratio) were calculated in the anteroposterior, mediolateral, and anteroposterior-mediolateral planes

**Results:**

Statistical analysis showed greater postural sway in robot-assisted conditions compared to Free. Mean distance increased from 1.7 (SD 0.6) cm in Free to 2.4 (SD 0.6) cm in RF (*P*<.001) and 2.3 (SD 0.6) cm in RP (*P*<.001). Mean velocity increased from 2.9 (SD 0.9) cm/s in Free to 4.3 (SD 1.4) cm/s in RF and RP. Confidence ellipse area increased from 7.6 (SD 4.1) cm^2^ in Free to 24.9 (SD 14.2) cm^2^ in RF and 23.1 (SD 13.4) cm^2^ in RP. Sway area increased from 1.5 (SD 0.7) cm^2^/s in Free to 2.9 (SD 1.2) cm^2^/s in RF and RP. Nonlinear metrics revealed lower recurrence rates in robot-assisted conditions, decreasing from 0.31 (SD 0.08) in Free to 0.2 (SD 0.08) in RF and 0.2 (SD 0.04) in RP in the anteroposterior-mediolateral plane (*P*<.001), from 0.33 (SD 0.08) in Free to 0.28 (SD 0.07) in RF (*P*=.02) and 0.16 (SD 0.03) in RP (*P*=.007) in the mediolateral direction, and from 0.36 (SD 0.07) in Free to 0.3 (SD 0.06) in RF (*P*=.009) and 0.26 (SD 0.03) in RP (*P*<.001) in the anteroposterior direction. Determinism remained stable (values close to 1), leading to higher determinism-to-recurrence ratios for robot-assisted conditions, increasing from 3.41 (SD 0.87) in Free to 5.41 (SD 1.69) in RF and 5.51 (SD 1.11) in RP in the anteroposterior-mediolateral plane (*P*<.001), from 3.11 (SD 0.63) in Free to 3.64 (SD 0.72) in RF (*P*=.02) and 3.92 (SD 0.48) in RP (*P*=.007) in the mediolateral direction, and from 2.82 (SD 0.48) in Free to 3.47 (SD 0.57) in RF (*P*=.009) and 3.46 (SD 0.45) in RP (*P*<.001) in the anteroposterior direction. No significant differences were found between the robot-assisted conditions.

**Conclusions:**

Interaction increases postural sway, indicating reduced stability and higher physical demand. This could reflect impaired balance or adaptation. Nonlinear analysis reveals that postural control remains structured. Results also suggest that the mere presence of the cobot is the primary driver of these postural changes.

## Introduction

Industry 4.0 has brought transformative changes to manufacturing, emphasizing the integration of industrial robots to minimize workers’ involvement in repetitive tasks [1]. Robots are now widely used in industrial settings to assist or replace workers in physically demanding tasks, offering benefits such as lower error rates, higher productivity, lower costs, and improved product quality. Recent advancements in robotics have also facilitated closer interactions between humans and robots, giving rise to human-robot collaboration (HRC), which has become a key focus in robotics research [2-4]. In HRC, humans and robots work side by side toward shared goals, engaging in continuous interactions where they adapt and respond to each other’s actions [5]. This synergy enhances efficiency and productivity, as human abilities complement robotic precision, strength, and endurance [6]. However, while HRC helps both workers and companies, its impact on physical and cognitive well-being requires careful assessment.

Numerous studies have explored how HRC affects workers, focusing on various aspects such as ergonomics, cognitive workload, safety, and overall well-being. Evaluating physical and mental demands in collaborative settings has been highlighted as a key factor [7], as ergonomic risk factors and comfort directly impact workers’ long-term health [8,9]. Although collaborative robots (cobots) aim to reduce physical strain by assisting workers in repetitive or strenuous tasks, they do not completely prevent work-related musculoskeletal disorders (WMSDs), which may arise from improper postures or behaviors [10]. WMSDs are a major concern in industries with manual labor and repetitive motions, potentially leading to chronic pain, reduced productivity, and long-term health problems [11,12]. When work tasks are performed in quasi-static conditions, direct measures of posture could reveal insights into the stability and balance of workers and can thus be used to improve ergonomics during HRC [13]. Poor or sustained postures can cause musculoskeletal imbalances, increased strain on joints and muscles, and altered movement patterns over time [14]. Posturography data could help evaluate musculoskeletal load, identify ergonomic risk factors, and develop strategies to mitigate them.

Traditionally, sensory-motor and cognitive mechanisms involved in balance control are studied using conventional center of pressure (COP) measures. COP represents the point where the resultant ground reaction force is applied during standing or movement, and its displacement over time provides valuable insights into postural stability and balance. Standard COP metrics include mean amplitude and mean distance (reflecting the extent of the postural sway), mean velocity (representing the speed of postural adjustments), and confidence ellipse area and sway area (characterizing the distribution of the sway) [15]. These parameters are crucial for understanding how the body maintains equilibrium, particularly during tasks that involve dynamic movement or changes in posture. Additionally, variations in COP data can help identify deviations from normal posture, which may indicate potential risks for musculoskeletal discomfort or injury.

However, some researchers have argued that traditional analyses of the spatial dynamics of postural activity are insufficient to fully understand the mechanisms underlying postural control and, in particular, cannot provide a complete understanding of stability and instability in the control of posture [16,17]. These measures offer an overall view of postural sway and may be linked to the adopted muscular strategy, but they do not directly refer to the adopted control strategy or to the attentional demand allocated to the control of balance. Specifically, these traditional parameters primarily focus on linear characteristics of postural control and may not fully capture the complexity of human balance regulation. This limitation suggests that nonlinear analysis could complement traditional measures of the extent and distribution of postural sway, offering a more comprehensive perspective on postural dynamics [18]. Deepening understanding of the mode of control and its underlying mechanisms has encouraged researchers to call for more powerful and relevant techniques of analysis [19].

Among the indicators recently introduced to capture distinct aspects of the nonlinear temporal dynamics of time series data, recurrence quantification analysis (RQA) has been hypothesized as a viable method to capture aspects linked to balance maintenance [20-26]. RQA describes the temporal structure of a dynamic system by quantifying the number and duration of repeated states [27]. Crucially, RQA does not merely describe the magnitude or variability of movement, as traditional COP metrics do, but reveals how postural adjustments evolve. This includes capturing aspects such as the repetitiveness of the displacement trajectory, the complexity of the control strategy, and the system’s predictability or adaptability [18], also in conditions where balance maintenance is challenged by the presence of repetitive supra-postural tasks [28]. Some of the most commonly used RQA parameters are the relative amount of recurrence and determinism [18].

Hence, traditional COP parameters offer a general overview of gesture characteristics, while RQA provides complementary insight by capturing the temporal structure and regularity of postural adjustments. Accordingly, this study combines both types of metrics to assess postural control and biomechanical risk during the execution of manipulatory tasks performed with and without robotic assistance. The experimental task followed a structured sequence consisting of rest, object grasping, object manipulation, object release, and rest. For this analysis, we focused only on the manipulation phase, when the participant actively interacted with the object, while grasping, release, and rest periods were excluded. This approach allows for a specific assessment of postural control precisely during sustained object handling. Unlike previous studies [13,29], which focused on sequential task execution with a cobot, this work examines a parallel task configuration, where the human and the cobot interact simultaneously, sharing the same space and concurring on the same task [30], thus introducing fundamentally different interaction dynamics. Accordingly, the goal of this work is twofold: first, to investigate whether the assistance of the cobot influences postural control and musculoskeletal load compared to performing the task alone; and second, to explore whether varying the mode of robotic collaboration within parallel tasks elicits distinct biomechanical responses. Ultimately, this approach aims to facilitate real-time monitoring of workers’ physical strain and support the development of ergonomic strategies to reduce WMSDs and enhance workplace safety.

## Methods

### Experimental Setup

**Figure 1.**
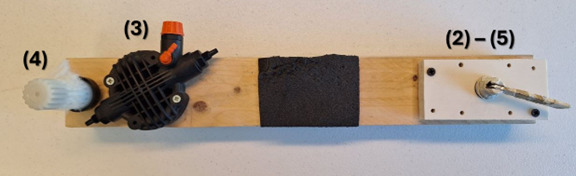
Object designed to be manipulated in the task. The numbered labels identify the items to be manipulated at each corresponding step of the task sequence.

The experimental setup was designed using a 2 × 1 m table on which a cobot (Franka Emika Panda, Munich, Germany) was positioned opposite the participant, who stood in an upright posture, to simulate a collaborative workbench environment. Participants were instructed to cyclically perform a manipulation task using a custom-designed tool (Figure 1), specifically developed to simulate common and functionally relevant manipulation activities. The tool is a wooden beam equipped with 4 interactive mechanisms: a white knob, a rotating component with a high-positioned orange knob, a black padded section designed for grip, and a white lever mounted on a rectangular structure.

The manipulation task consisted of the following steps: (1) take the object, (2) open the lever on the right by rotating it 90° counterclockwise, (3) rotate the high-positioned orange knob until it reaches the end of its stroke, (4) unscrew the white knob on the left for 2 full turns, (5) close the lever that was opened at step 2, and (6) return the object to its original position. After completing this sequence, participants rested with their arms at their sides. Following a brief pause, they independently decided when to initiate the next cycle of manipulation. The experiment included three conditions:

1. Free: the participant performed the task independently. The object was positioned on an elevated support on the right side of the table for easy access. Using their right hand, the participant grasped the object and executed a predefined sequence of movements. The task was considered complete once the object was returned to its initial position.
2. Robot Free (RF): the cobot assisted the participant by securely holding the object at its central part, simulating a “gravity compensation” condition, thus minimizing the load the participant was required to support. In this condition, the cobot could move freely in response to the participant’s needs. The task began with the cobot holding the object, after which the participant approached and performed the required sequence of movements. The task ended when the participant guided the cobot back to its starting point on their right.
3. Robot Plane (RP): similar to the RF condition, the cobot securely held the object at its central part. However, in this condition, the robot’s movement was constrained to a virtual horizontal plane, locking the object’s vertical movement and allowing free movement in other directions. The task shared the same starting and ending configurations as the RF condition. The height of the simulated plane was kept fixed for all experiments, regardless of the participant.

**Figure 2.**
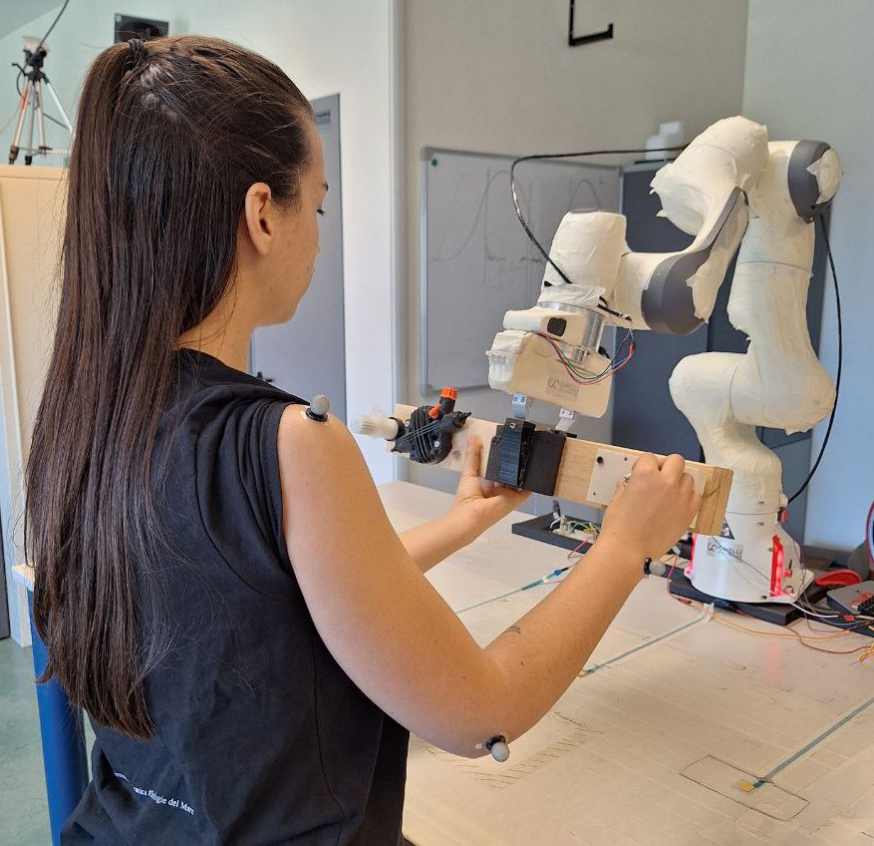
Example of a manipulation task with robotic assistance.

The inclusion of 2 distinct robot-assisted conditions was motivated by the hypothesis that varying the mode of collaboration could elicit different effects on human motor behavior and postural control. Specifically, the RP condition implements a gravity-compensation scenario in which the object is virtually weightless, and its vertical position is fixed. This constraint results in a different interaction with the cobot, while in the RF condition, participants need to continuously support and stabilize the cobot with 1 hand while performing the task with the other. In the RP condition, they can rely on the cobot’s fixed height, potentially leading to the adoption of different motor strategies. Moreover, the inability to move the robot vertically may require participants to adapt their posture or reposition themselves to comfortably perform specific movements. This distinction between RF and RP thus allows the researchers to examine how different kinematic constraints and interaction modes with the cobot influence postural control and task execution. An illustrative photo of an individual performing the task under robot-assisted conditions is shown in Figure 2.

Participants were free to move naturally in all conditions, with the only requirement being the use of their right hand to grasp the object. Each condition was repeated 3 times, with each trial consisting of 5 repetitions of the manipulation task. The trials were conducted in a randomized order to minimize potential learning effects and ensure natural task execution.

### Data Acquisition and Processing

Fourteen healthy right-handed male participants took part in the study (age: mean 26, SD 3 years; height: mean 177, SD 6 cm; and weight: mean 71, SD 7 kg). Participants were recruited based on their nonfamiliarity with the task and the absence of any known musculoskeletal, neurological, or visual impairments. The study was approved by the local ethical committee of Roma Tre University.

Two 6-component force platforms (BTS P-6000 and BTS Bioengineering) were positioned under the participants’ feet to record ground reaction forces and to derive the COP coordinates. Force data were collected at a sampling frequency of 500 Hz. Each platform was positioned under 1 foot. Additionally, a marker-based optoelectronic system (BTS SMART-DX 6000 and BTS Bioengineering) was used to segment and identify each task repetition, with passive markers placed at key anatomical landmarks. These kinematic data were acquired using a sampling frequency of 250 Hz. Force platform data were then resampled at the same rate.

### Data Processing and Analysis

Trajectories of the COP for each of the lower limbs (ie, COP-right and COP-left) were extracted from the force platform data. The total COP was then obtained as the weighted average between COP-right and COP-left, and its displacement was defined by both the anteroposterior and the mediolateral coordinates with respect to a reference system centered between the 2 force plates. Cycles of the movement were identified using motion capture data: segmentation was made by detecting the time instant when the marker on the right wrist reached the maximum rightward excursion in the mediolateral direction, indicating the completion of a repetition. Only manipulation phases were extracted for the analysis, as the focus was specifically on these active interactions, excluding the other phases (ie, rest, object pick-up, and object release).

Four COP-derived sway parameters were selected to evaluate postural stability and quantify balance changes: mean distance, mean velocity, 95% confidence ellipse area, and sway area. These metrics were chosen based on their ability to capture distinct yet complementary aspects of postural control and stability. Mean distance quantifies the extent of postural displacement, reflecting the range of movement and how far the COP deviates from a reference point. Mean velocity measures the speed of postural displacement, indicating how quickly the individual adjusts their posture. The ellipse area captures the spread of the COP, providing insights into postural control by assessing variability and dispersion. This metric was preferred over the confidence circle area, as the elliptical estimate accounts for the directionality of sway—a relevant feature when COP trajectories are not uniformly distributed but follow a predominant path [13]. The sway area provides a measure of the extent of postural instability during task execution. For each cycle of the task repetition in each experimental condition, these parameters were calculated as defined in [30]. Mean values were then computed and subjected to inferential statistical analysis to assess potential differences across conditions.

**Figure 3.**
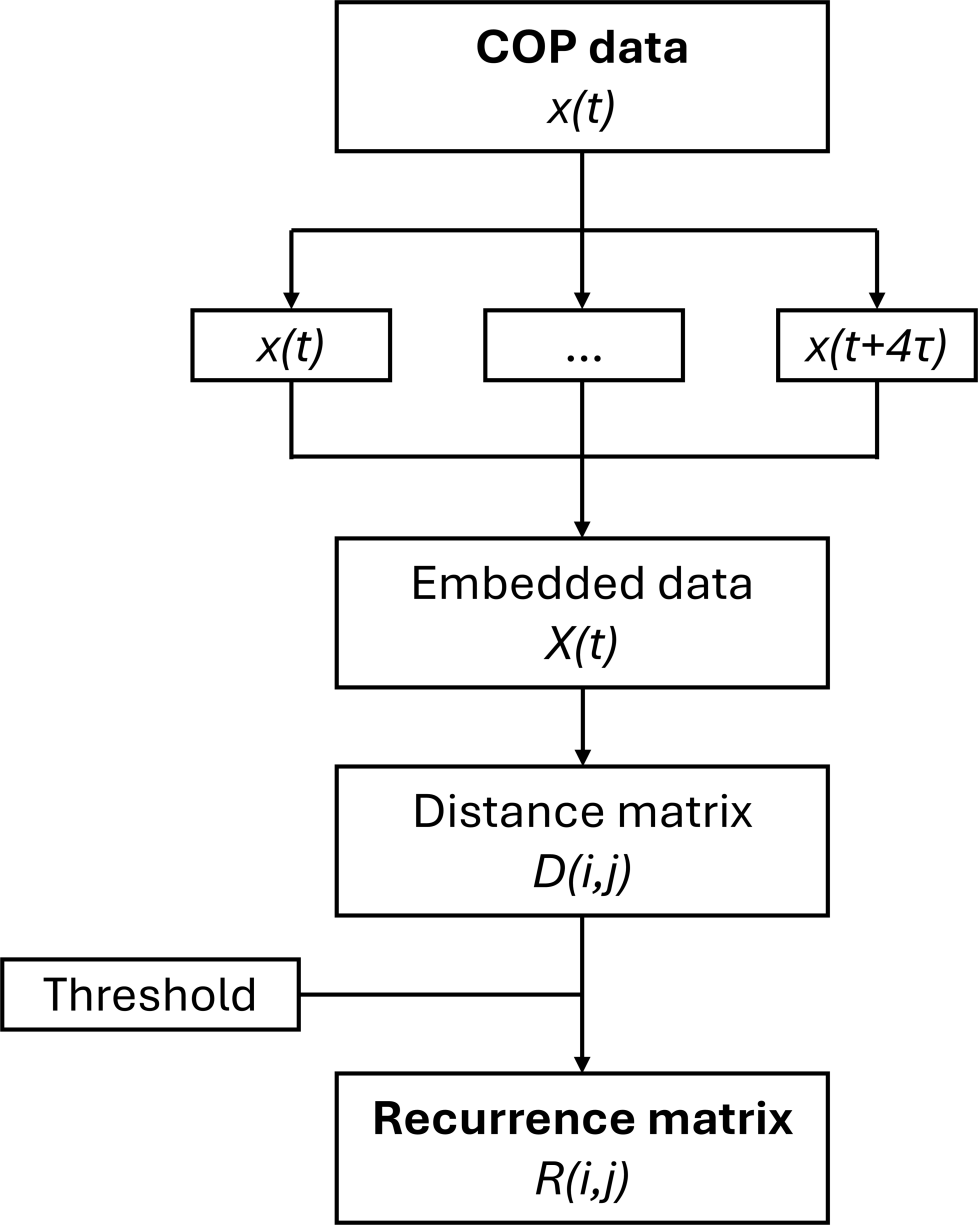
Flowchart for the embedding of center of pressure (COP) data and the extraction of the recurrence matrix.

In addition to these conventional analyses, an RQA was performed to explore the underlying dynamical patterns of postural sway. These measures provide insight into the temporal organization, predictability, and adaptability of postural control—aspects that are especially relevant in dynamic and interactive tasks—and are not influenced by participants’ anthropometric characteristics. To investigate dynamics on a cycle-based time reference, the time delay was set to the average length of the cycle evaluated for each trial. The processing steps adopted for extracting the recurrence maps, similar to those used in [29], are reported in Figure 3.

RQA was carried out on the radial component of the COP, as well as on the anteroposterior and mediolateral components alone. Specifically, 3 parameters were calculated to analyze the dynamics of postural sway [29]. The recurrence rate (REC), also known as the density of recurrence points in the recurrence plot [18], quantifies the percentage of recurrent states in the COP trajectory, reflecting the overall stability of postural sway. Determinism indicates the proportion of those recurrence points that form structured diagonal lines, which is associated with the predictability and regularity of sway patterns. The ratio, defined as the ratio between determinism and REC, represents the extent to which the recurring patterns are deterministic in nature.

### Statistical Analysis

Normality of sway parameters and RQA indicators was assessed using the Shapiro-Wilk test. Since at least 1 of the distributions of each parameter was not normal, nonparametric statistical analyses were applied. To evaluate differences in both traditional and RQA-based parameters across the 3 experimental conditions, a Friedman test was performed. When a significant main effect was found, pairwise comparisons were conducted using the Wilcoxon rank-sum test. The significance level was set to 0.05, 0.01, and 0.001 (indicated by *, **, and ***, respectively). Effect sizes were calculated to quantify the magnitude of observed differences and are reported as correlation coefficients (*r*), with thresholds of 0.1‐0.3, 0.3‐0.5, and >0.5 denoting small, medium, and large effects, respectively.

### Ethical Considerations

Before participation, all participants provided informed consent, and the study was approved by the Ethical Commission of Roma Tre University (BRIC2022-CoRoMan approval r.01-24/06/2025). Participants did not receive any compensation for the participation, and all data was anonymized for maintaining privacy and confidentiality of the information.

## Results

### Linear Metrics

**Figure 4.**
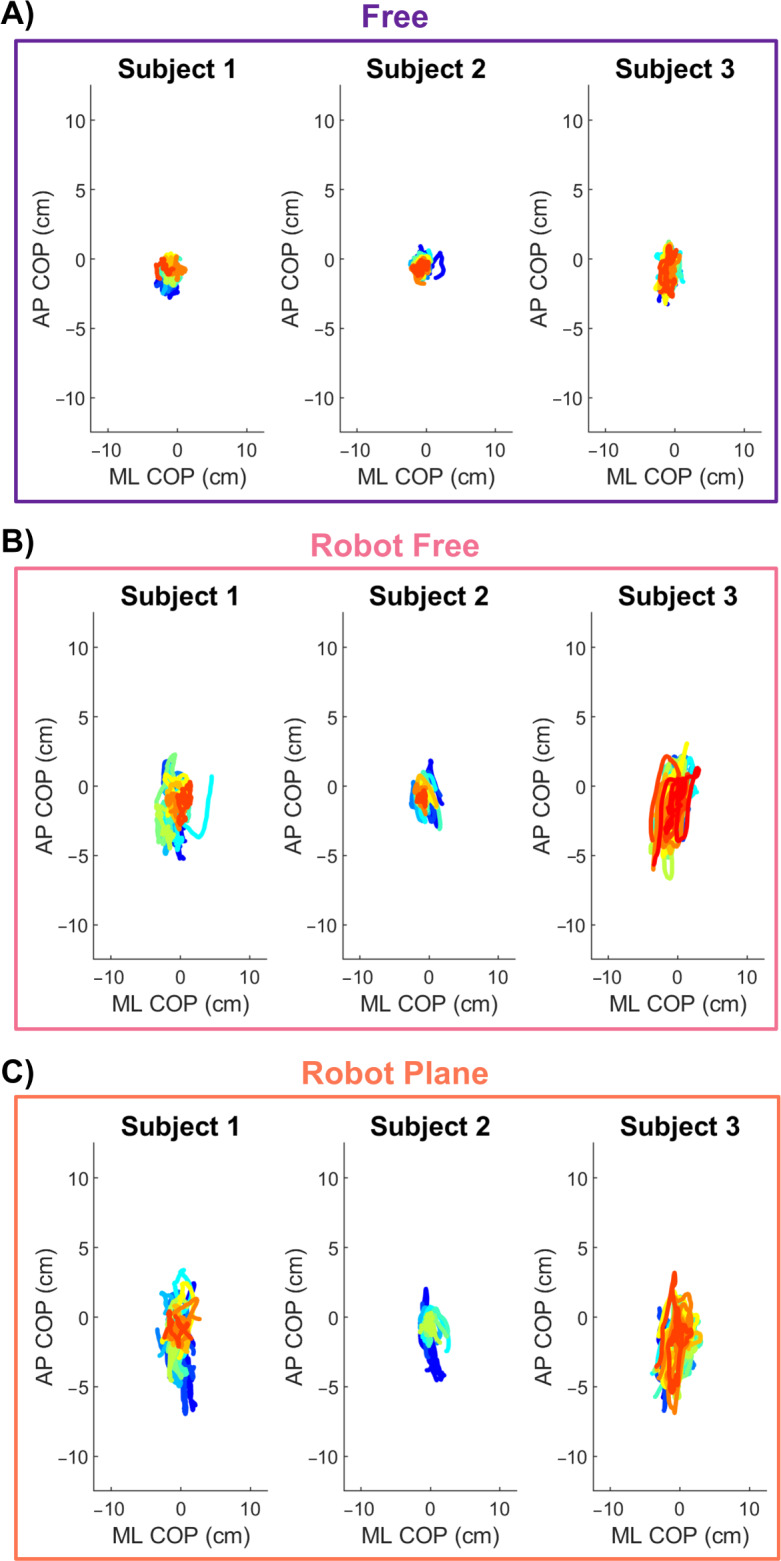
Center of pressure (COP) trajectories of 3 representative participants across the 3 experimental conditions: (A) Free, (B) Robot Free, and (C) Robot Plane. Each panel displays individual COP traces, with colors indicating different task repetitions. AP: anteroposterior; ML: mediolateral.

Figure 4 shows the COP trajectories of 3 representative participants in the 3 experimental conditions.

In the Free condition (Figure 4A), COP trajectories exhibit a compact pattern for all considered participants, extending roughly 5 cm in both mediolateral and anteroposterior directions. Paths remain well-contained within a small spatial envelope, showing limited variability across cycles. In the RF condition (Figure 4B), COP trajectories display a broader pattern compared to those observed in the Free condition (Figure 4A), particularly along the anteroposterior axis. The range of displacement remains around 5 cm in the mediolateral direction but increases to approximately 15 cm in the anteroposterior direction. Intrasubject variability across cycles remains limited. Similar to what is observed in the RF condition (Figure 4B), COP trajectories in the RP condition (Figure 4C) exhibit a broader pattern compared to those in the Free condition (Figure 4A), particularly along the anteroposterior axis. Displacement in the mediolateral direction remains around 5 cm, while in the anteroposterior direction, it increases to approximately 10 cm. Intrasubject variability across cycles remains generally limited. These patterns were observed across all participants, with limited intersubject variability, demonstrating the consistency of the findings. Refer to multimedia appendices for the complete set of COP traces of all participants and all experimental conditions (refer to Multimedia Appendix 1 for Free, Multimedia Appendix 2 for RF, and Multimedia Appendix 3 for RP).

**Figure 5.**
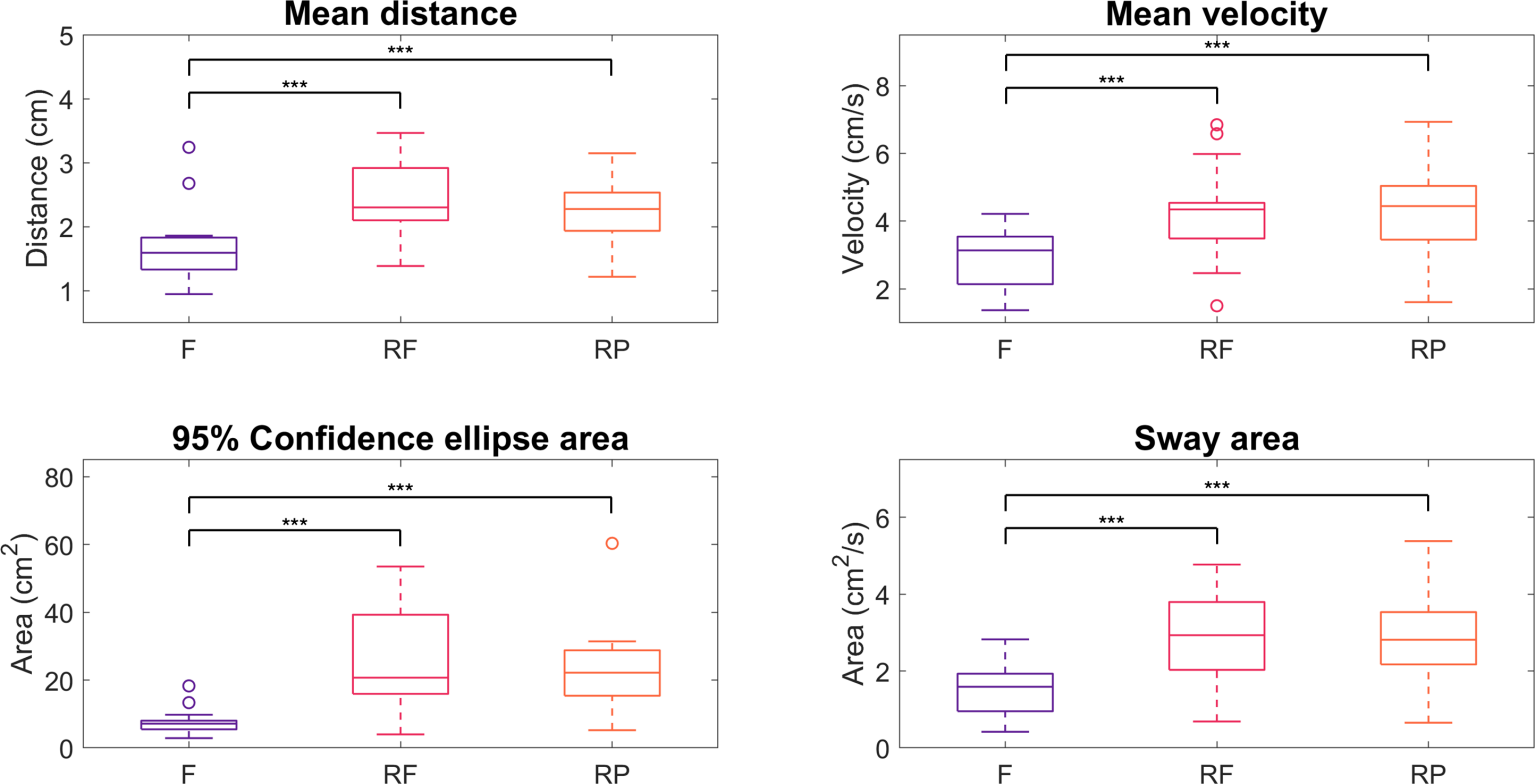
Distribution of sway parameters, differentiated by experimental conditions. “***” denotes *P*<.001 as obtained from inferential statistics. F: Free; RF: Robot Free; RP: Robot Plane.

The qualitative differences observed in COP trajectories are supported by the statistical analysis of sway parameters, as illustrated in Figure 5. The results reveal significant differences between the Free condition and both robot-assisted conditions, with the latter exhibiting consistently higher values across all parameters. In contrast, no significant differences were found between the 2 robot-assisted conditions for any of the parameters analyzed. Mean distance increased from 1.7 (SD 0.6) cm in Free to 2.4 (SD 0.6) cm in RF (*P*<.001; *r*=0.86) and 2.3 (SD 0.65) cm in RP (*P*<.001; *r*=0.86). Mean velocity increased from 2.9 (SD 0.9) cm/s in Free to 4.3 (SD 1.4) cm/s in RF and RP (*P*<.001; *r*=0.88). Confidence ellipse area increased from 7.6 (SD 4.1) cm^2^ in Free to 24.9 (SD 14.2) cm^2^ in RF (*P*<.001; *r*=0.88) and 23.1 (SD 13.4) cm^2^ in RP (*P*<.001; *r*=0.88). Sway area increased from 1.5 (SD 0.7) cm^2^/s in Free to 2.9 (SD 1.2 cm^2^/s) in RF and RP (*P*<.001; *r*=0.88).

### Nonlinear Metrics

**Figure 6.**
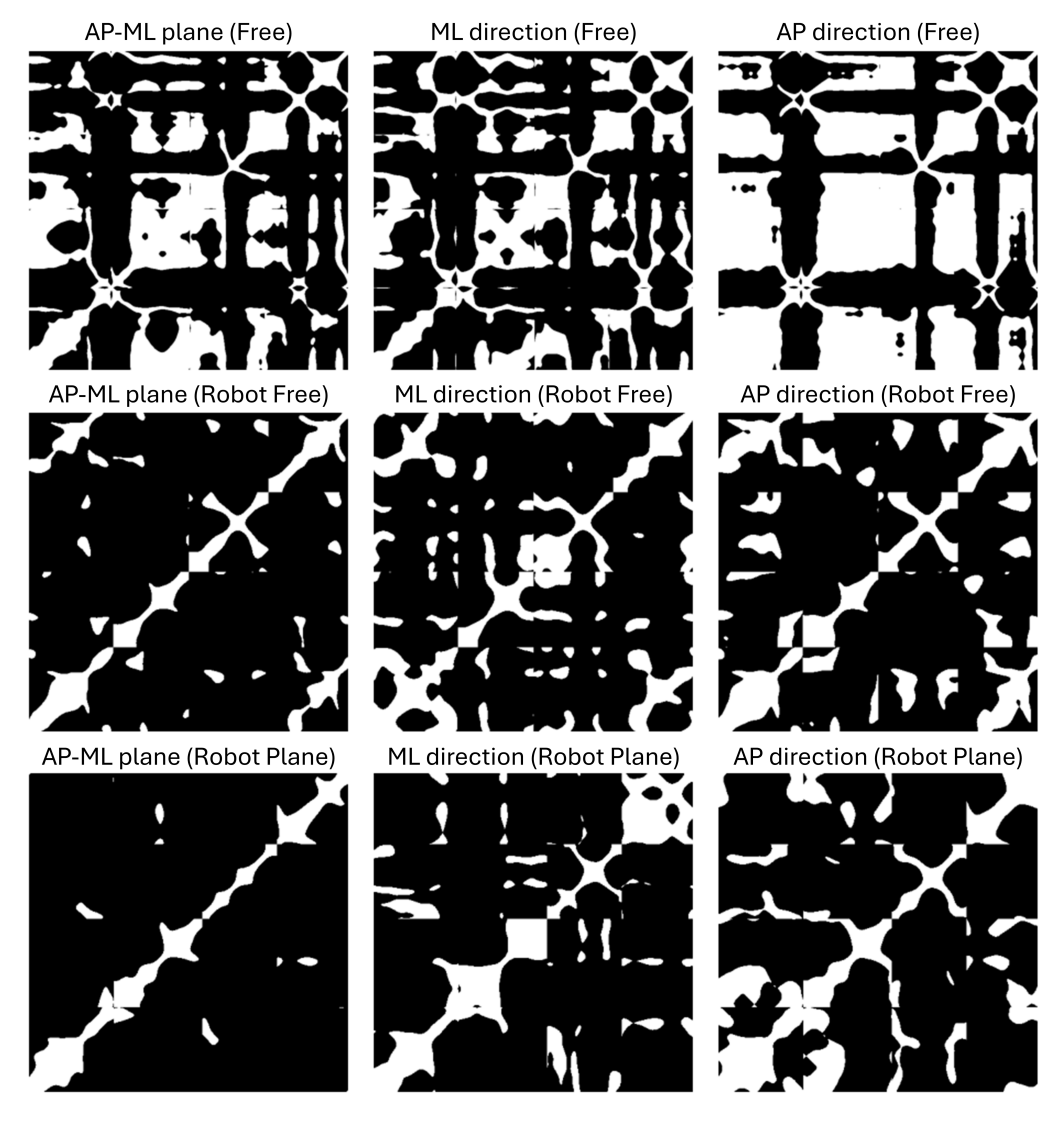
Sample recurrence maps, relative to 1 exemplary participant, in different trials and for different directions. AP: anteroposterior; ML: mediolateral.

An example of the RQA recurrence maps obtained from a subset of the cycles relative to 1 exemplary participant is reported in Figure 6. A consistent pattern emerges across both robot-assisted conditions, marked by a greater density of points along the principal diagonal line in the recurrence maps compared to the Free condition. The Free condition exhibits a more pronounced checkered structure, reflecting the prevalence of recurrent quasi-stationary states.

**Figure 7.**
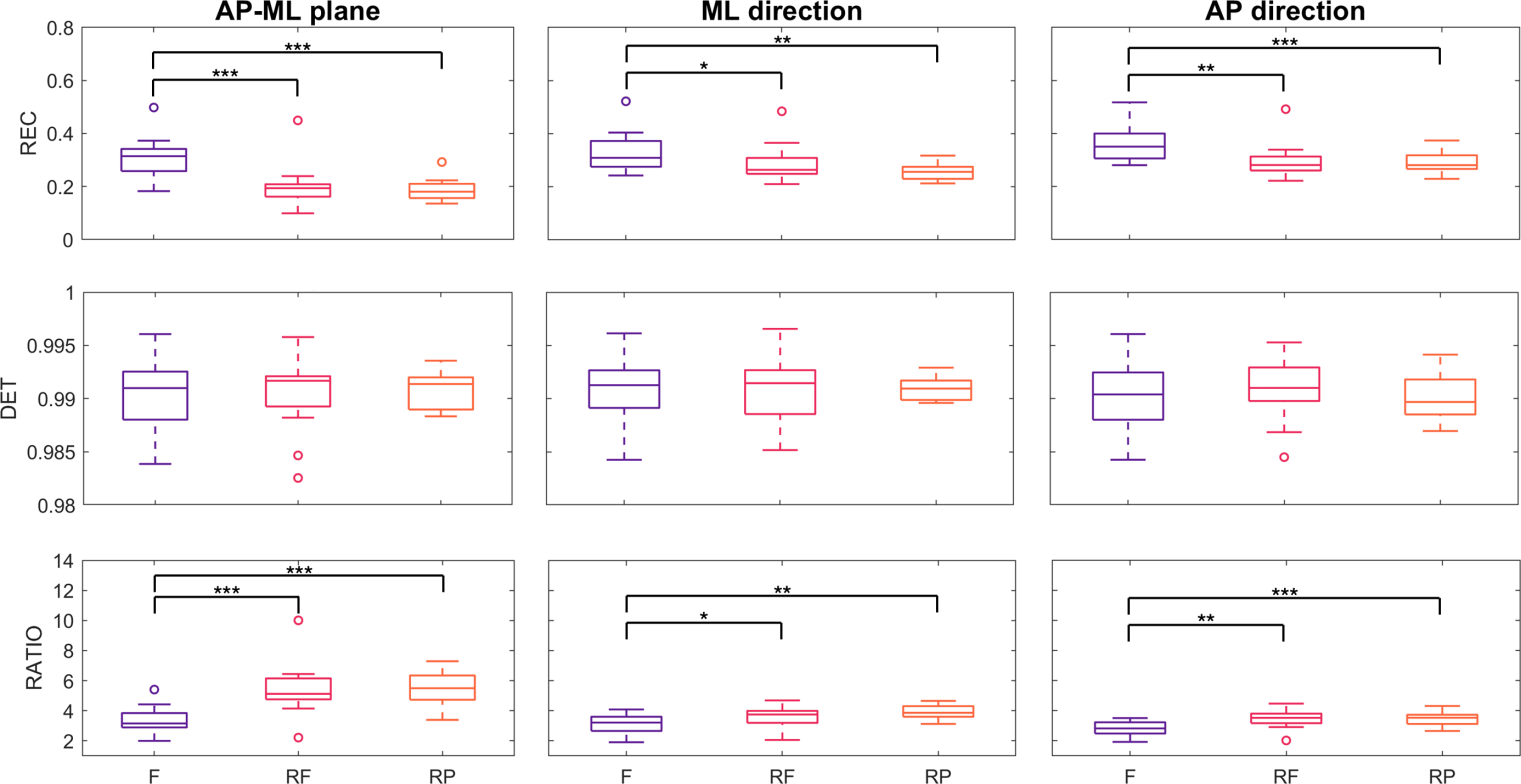
Distribution of recurrence quantification analysis (RQA) parameters, differentiated by experimental conditions. “*,” “**,” and “***” denote *P*<.05, *P*<.01, and *P*<.001, respectively, as obtained from inferential statistics. AP: anteroposterior; DET: determinism; F: Free; ML: mediolateral; RATIO; REC: recurrence rate; RF: Robot Free; RP: Robot Plane.

These qualitative and specific observations are supported by the quantitative results shown in Figure 7, which presents boxplots of the RQA-based parameters for all participants. Specifically, the REC is significantly lower in both robot-assisted conditions. REC decreased from 0.31 (SD 0.08) in Free to 0.2 (SD 0.08) in RF (*P*<.001; *r*=0.88) and 0.2 (SD 0.04) in RP (*P*<.001; *r*=0.88) in the anteroposterior-mediolateral plane; it decreased from 0.33 (SD 0.08) in Free to 0.28 (SD 0.07) in RF (*P*=.02; *r*=0.65) and 0.26 (SD 0.03) in RP (*P*=.007; *r*=0.75) in the mediolateral direction; it decreased from 0.36 (SD 0.07) in Free to 0.3 (SD 0.06) in RF (*P*=.009; *r*=0.75) and 0.26 (SD 0.03) in RP (*P*<.001; *r*=0.86) in the anteroposterior direction. On the other hand, determinism remains largely unchanged. Consequently, the ratio between determinism and REC is significantly higher during robotic assistance, reflecting a shift toward more periodic and structured postural control strategies when the cobot is engaged. This ratio increased from 3.41 (SD 0.87) in Free to 5.41 (SD 1.69) in RF (*P*<.001; *r*=0.88) and 5.51 (SD 1.11) in RP (*P*<.001; *r*=0.88) in the anteroposterior-mediolateral plane; it increased from 3.11 (SD 0.63) in Free to 3.64 (SD 0.72) in RF (*P*=.02; *r*=0.6) and 3.92 (SD 0.48) in RP (*P*=.007; *r*=0.75) in the mediolateral direction; it increased from 2.82 (SD 0.48) in Free to 3.47 (SD 0.57) in RF (*P*=.009; *r*=0.75) and 3.46 (SD 0.45) in RP (*P*<.001; *r*=0.86) in the anteroposterior direction. It is worth noting that the determinism values observed here are higher than those reported in [28], which can be attributed to our use of a lower-dimensional embedding space and different threshold settings in the RQA procedure.

## Discussion

### Principal Findings

The analysis of the COP trajectories shows a greater extent along the anterior-posterior direction in robot-assisted conditions with respect to the Free condition, possibly indicating a higher number of oscillations of the body when the cobot intervenes in the task execution. This could suggest that participants tend to deviate from their postural stability, potentially compromising the overall efficiency and coordination of the kinematic chain during task execution. The absence of an evident difference between COP paths in the RF and RP conditions may indicate that the mode of collaboration with the cobot does not substantially alter the way robotic assistance affects postural stability.

Suggestions resulting from these qualitative observations align with quantitative results from the analysis of traditional COP parameters. Indeed, higher values of all parameters were found in robot-assisted conditions compared to the Free condition, suggesting altered postural dynamics in response to robotic assistance, regardless of the mode of collaboration with the cobot.

Higher mean distance may result from robotic constraints altering postural stability and strategy. Since mean distance is commonly interpreted as an indicator of postural control effectiveness [31], higher values are typically associated with a reduced ability to maintain a stable upright position [15]. Larger COP excursions have also been linked to reduced postural control [32] and balance impairments [33-35].

Higher mean velocity may suggest increased postural control effort and adjustments, likely due to imposed constraints from robotic assistance. Faster COP movements are often interpreted as indicators of increased demand for active postural control [31] or postural instability [32,36], assuming that the mean distance remains constant. However, in our case, the mean distance also increases under collaborative conditions. This combined increase complicates the interpretation; rather than indicating instability alone, it may reflect an active and purposeful postural adaptation to the new collaborative context.

A larger confidence ellipse area usually indicates higher COP exploratory behavior in postural sway, which may represent an adaptive response to the altered task dynamics under robotic assistance. This measure is in some cases associated with reduced balance control [37] and difficulty in maintaining stability during upright standing [38].

A greater sway area reflects increased movement, possibly in response to the higher postural control demands imposed by robot interaction. Since sway area is a hybrid measure capturing both the interaction between postural control activity and stability level [31], larger values are usually interpreted as a sign of decreased stability [39].

Contrary to our initial hypothesis, the analysis revealed no significant differences between the RF and RP conditions across all the parameters. While we expected that constraining the robot’s movement might lead to changes in postural dynamics, the results suggest otherwise: the mere presence of robotic support, regardless of the collaboration mode, plays a dominant role in shaping participant responses. Indeed, the assistance provided by the cobot in both conditions may be sufficient to modulate the demands of the task, leading to similar adaptations.

Overall, COP tends to travel more (higher mean velocity and, to a certain extent, sway area) and further (mean distance and ellipse area) when robotic assistance is provided. In general, larger COP excursions are associated with reduced balance control, indicating a decreased ability to remain in a smaller (and typically more stable) area during task repetitions [13]. This may suggest that the cobot influences task execution, which is particularly relevant given that increased COP excursions are associated with the development of WMSDs [40]. From this perspective, the Free condition appears to allow the most effective postural control and imposes the lowest associated risk, as evidenced by more stable and restricted sway patterns.

These findings might be the result of an alteration of the body schema, an integrated and dynamic internal representation of the body and its surrounding space [41], continuously shaped and updated through sensory and social interactions [42]. Efficient movement in space requires both localizing objects in extrapersonal space and maintaining a constantly updated status of body shape and posture [43]. The body schema reflects how the body interacts with its environment, and it is highly plastic, adapting to novel tools or altered body representations [44]. Here, the cobot holding the object may have challenged this schema, leading to difficulty in incorporating the cobot interaction and thereby potentially explaining the increased postural sway. Indeed, recent works may suggest that interactions with robots and virtual agents can disrupt or extend embodiment, requiring new sensorimotor adaptations [45-50].

Nonlinear results further support this interpretation. A lower REC in robot-assisted conditions may suggest a more variable or exploratory balance strategy, as postural states repeat less frequently. This indicates a shift away from stable, habitual postural control patterns toward more dynamic adjustments, potentially reflecting the fact that the cobot is not yet fully integrated into the participant’s body schema. In this context, participants may need to continuously recalibrate their posture in response to the cobot’s actions, rather than relying on familiar strategies. This interpretation aligns with the idea that technological artifacts reshape bodily experience and require new sensorimotor adaptations [43,48,51,52]. Notably, REC values are comparable across the 2 robot-assisted conditions, suggesting that the mode of interaction does not further alter the degree of postural control. In other words, the presence of the cobot itself introduces a fundamental shift in balance strategy, but the specifics of the interaction mode may have a limited additional effect on this aspect of postural control.

Comparable determinism values across all conditions indicate that, despite the increased postural sway and lower recurrence observed in the robot-assisted conditions, the postural responses remain structured rather than random. This suggests that participants are still using organized control strategies, even if these strategies are more adaptive and less repetitive. This reflects a balance between flexibility and structure: while the presence of the cobot necessitates ongoing adjustments, participants appear to adopt consistent patterns, rather than reacting in a chaotic or unpredictable manner. This might be related to Black’s critique of the “natural interface” assumption [51]: the robot-object system does not immediately integrate as a seamless extension of the body, but does not cause complete instability either. Although the cobot may provide sufficient task stability, its presence is not yet fully integrated into the body schema, requiring continuous movement adjustments while maintaining a coordinated response. However, confirming this interpretation would require long-term longitudinal studies to assess whether such adaptations persist, diminish, or evolve.

Importantly, as observed with conventional COP measures, no significant differences were found between the 2 robot-assisted conditions across all RQA-based parameters, further indicating that the specific mode of parallel collaboration did not substantially affect overall postural outcomes. Thus, RQA findings suggest that interaction with the cobot challenges postural control without introducing randomness in balance control. The presence of the cobot appears to prevent full postural automatization, requiring individuals to engage in ongoing adjustments rather than relying on familiar movement patterns.

These results are also consistent with broader research on tool use, which demonstrates that tools can alter both perceptual and motor processes in healthy individuals as well as those with brain damage [41,43,52,53]. Effective tool use involves more than simple physical manipulation; it requires recognizing the object as a tool, knowing how to interact with it, and understanding the outcomes of its use [54,55]. These processes may be difficult to fully integrate into the body schema, particularly when new dynamics are introduced that the body has not yet adapted to, such as those posed by a cobot.

Overall, our results suggest that robotic assistance affects postural control and may challenge the body schema. However, interpretations in terms of motor learning, tool use, or body schema adaptation remain speculative at this stage. The observed changes in linear and nonlinear metrics indicate postural adjustments, yet it is unclear whether these reflect short-term responses or longer-term learning. Further research is needed to clarify how these adaptations develop over time.

### Limitations

Although this study offers meaningful evidence regarding the effects of cobots on human postural control, several limitations should be considered when interpreting the results.

First, the research was conducted in a controlled laboratory setting using a standardized manipulation sequence, a simulated workbench, and a custom tool. The task sequence was carefully designed to closely resemble typical industrial manipulation operations and to capture key aspects of HRC, such as coordination with a robotic partner, repetitive handling, and precise posture adjustments in this context. While lab-based tasks cannot fully replicate the complexity and variability of real industrial scenarios, our design sought to approximate these conditions to ensure relevance to typical HRC environments. Future studies should incorporate diverse object types, task sequences, and interaction dynamics to further strengthen ecological validity and assess robustness across broader industrial contexts.

Second, the participant sample consisted of 14 healthy young adult males, and this limits generalizability to the broader industrial workforce. Different populations may respond differently in HRC contexts: older adults or individuals with musculoskeletal impairments might exhibit greater postural sway and slower movements, while sex-related differences in strength, flexibility, and neuromuscular coordination could alter movement strategies. These factors suggest that our findings capture only part of the variability present in industrial settings. At the same time, the large effect sizes observed in our analyses indicate that the identified effects are robust and not solely sample dependent. Moreover, when considering a broader population sample, we expect that, even though sway parameters may be affected by anthropometric characteristics, nonlinear indicators would reasonably maintain similar variations. Future research should, however, include participants of different ages, sexes, and health conditions to improve outcomes.

Moreover, the study focused on short-term cobot interactions, leaving open questions about the long-term effects of repeated exposure, which might influence postural stability and increase the risk of developing musculoskeletal disorders. Investigating whether initial adaptations persist, diminish, or intensify over time, by incorporating more extended sessions, is therefore essential to clarify how postural control reacts or adapts to long-term exposure to HRC.

Finally, although RQA measures are computationally robust and have undergone rigorous validation, their novelty makes interpretation complex and context dependent [35]. As noted in the “Discussion” section, attributing the observed alterations in postural dynamics to body schema changes remains hypothetical and requires complementary methods (eg, kinematics, electromyography, and questionnaires on perceived effort) and longitudinal studies to support the evidence and strengthen causal inferences. This more comprehensive approach would reinforce the ergonomic perspective and offer a more precise understanding of both physical and mental demands in HRC scenarios.

### Conclusions

This study investigated how HRC influences postural control and musculoskeletal load during dynamic manipulation tasks performed in upright stance. Postural control was evaluated using traditional COP-derived linear metrics and nonlinear RQA indicators. Findings show that interaction with the cobot increases COP excursions and velocity, indicating higher postural demands and reduced stability compared to performing the task alone [56]. These effects likely stem from task-specific challenges, movement constraints imposed by the cobot, and adaptive neuromuscular system responses. RQA analysis revealed lower RECs but stable determinism, suggesting that postural adjustments become more variable yet remain structured, reflecting adaptive rather than random responses. No significant differences were observed between the 2 robot-assisted conditions, implying that the cobot’s presence, rather than its movement constraints, drives these postural changes.

Overall, these findings indicate that HRC tasks may challenge the body schema and require continuous sensorimotor adaptation, potentially influencing both task execution and balance strategies. From an ergonomic perspective, the results highlight that cobotic tasks can affect whole-body postural organization, even when local task execution remains unchanged. This supports the inclusion of postural control and postural dynamics as complementary indicators in ergonomic assessments of HRC, alongside conventional biomechanical measures. Such insights are critical for ergonomic cobot design, emphasizing the importance of offering assistance that supports, rather than disrupts, natural postural control. In industrial contexts, cobots have the potential to enhance safety, productivity, and ergonomics; however, their impact on postural stability must be carefully considered. Increased COP excursions have been linked to a higher risk of musculoskeletal disorders [13]; poorly designed cobot interactions could thus inadvertently increase physical strain. Conversely, when properly integrated, cobots could reduce the physical demands of repetitive tasks, enabling safer and more ergonomic working conditions. Optimizing cobot design and interaction strategies is therefore essential to ensure that cobots complement human capabilities, support natural movement, minimize physical strain, and promote sustainable, safer human-centered work environments.

## Supplementary material

10.2196/79930 Multimedia Appendix 1 Center of pressure (COP) trajectories for each participant during the Free experimental condition. Each color is associated with each task repetition.

10.2196/79930 Multimedia Appendix 2 Center of pressure (COP) trajectories for each participant during the Robot Free experimental condition. Each color is associated with each task repetition.

10.2196/79930 Multimedia Appendix 3 Center of pressure (COP) trajectories for each participant during the Robot Plane experimental condition. Each color is associated with each task repetition.

## Abbreviations

Cobot: collaborative robot
COP: center of pressure
HRC: human-robot collaboration
REC: recurrence rate
RF: Robot Free
RP: Robot Plane
RQA: recurrence quantification analysis
WMSD: work-related musculoskeletal disorder
