# Supplementary material for: Dynamic Balance Control and Postural Adaptation in Human-Robot Collaborative Manipulation: Within-Subject Experimental Study
Source: JMIR Hum Factors. 2026 Apr 23;13:e79930. doi: 10.2196/79930 (PMC13105445; doi:10.2196/79930)
Supplement: Multimedia Appendix 1 [file humanfactors-v13-e79930-s001.docx]

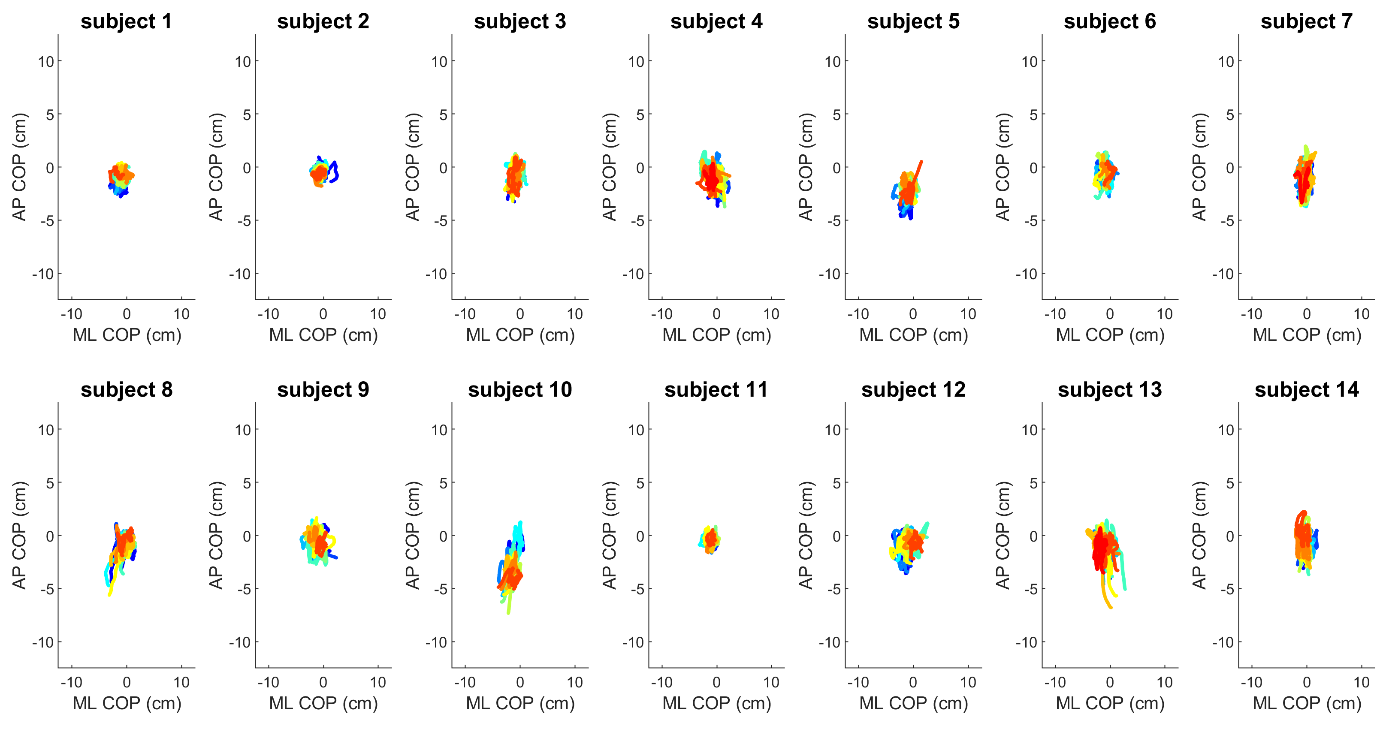


Figure 1. COP trajectories for each subject during the Free experimental condition. Each color is associated with each task repetition.
